# Unseen struggles: a narrative review of psychosocial and family outcomes following pediatric stroke

**DOI:** 10.3389/fpsyg.2026.1784867

**Published:** 2026-03-27

**Authors:** Samuela Tarantino, Martina Proietti Checchi, Michela Ada Noris Ferilli, Gabriele Monte, Alessandro Borrelli, Giuseppe Tiralongo, Massimiliano Valeriani

**Affiliations:** 1 Developmental Neurology Unit, Bambino Gesù Children's Hospital, Istituto di Ricovero e Cura a Carattere Scientifico (IRCCS), Rome, Italy; 2 Academy of Pediatrics, Tor Vergata University of Rome, Rome, Italy; 3 Systems Medicine Department, Tor Vergata University of Rome, Rome, Italy; 4 Translational Pain Neuroscience and Precision Medicine, CNAP, Department of Health Science and Technology, School of Medicine, Aalborg University, Aalborg, Denmark

**Keywords:** anxiety, children, depression, family, stress, stroke

## Abstract

Pediatric stroke results in complex sequelae that extend beyond motor and cognitive impairments to include significant psychological and psychosocial challenges for both affected children and their families. This narrative review synthesized peer-reviewed studies published in English between 2000 and November 2025, identified through searches in PubMed and Web of Science using keywords related to pediatric stroke and psychosocial outcomes. Eligible studies examined psychological, social, and family-related outcomes, and findings were integrated through a narrative synthesis due to substantial methodological heterogeneity. Internalizing symptoms-such as anxiety, depression, and post-traumatic stress disorder-commonly emerge and may persist long-term, even in cases of favorable neurological recovery. Parental psychological distress, particularly maternal anxiety, depression, and sense of guilt, together with family functioning, are closely associated with children's emotional and behavioral adjustment, reflecting a bidirectional relationship between caregiver and child wellbeing. Recovery after pediatric stroke should be conceptualized as a comprehensive process, requiring a multidisciplinary, family-centered framework incorporating psychological support that addresses not only motor and cognitive deficits but also emotional and psychosocial challenges. Future research and clinical efforts should focus on the development and implementation of accessible, empirically validated interventions aimed at enhancing resilience and long-term quality of life for patients and their families.

## 1 Introduction

Pediatric stroke is defined as a cerebrovascular event occurring between 20 weeks of fetal life and 18 years of age, resulting from an acute disruption of cerebral blood flow due to arterial ischemia, cerebral sinovenous thrombosis, or intracranial hemorrhage (Sporns et al., 2022). This interruption of perfusion leads to focal or global brain injury and may result in persistent neurological deficits, depending on the timing, location, and extent of the lesion. Although rare, it is a serious neurological condition. The most common subtypes include arterial ischemic stroke (AIS) and cerebral sinovenous thrombosis (CSVT). Literature-based data indicate that the birth prevalence of perinatal stroke ranges from approximately 1 in 1,000 to 1 in 3,000 live births, while the annual incidence of stroke in children is reported at 1.2–13 cases per 100,000 children per year (Srivastava and Kirton, 2021; Rawanduzy et al., 2022). Advances in acute care have significantly improved survival rates. However, many children continue to experience long-term sequelae that affect not only their physical development but also their daily functioning and quality of life (Friefeld et al., 2004, 2011; Ghotra et al., 2015; Svensson et al., 2022; Yvon et al., 2018; Li et al., 2021; Janas et al., 2023; Rivella et al., 2023a). Motor impairments, particularly hemiparesis, are among the most prevalent outcomes, limiting mobility and participation in daily activities (Yvon et al., 2018; Li et al., 2021; Svensson et al., 2022; Janas et al., 2023). Cognitive difficulties are also common, often involving attention, memory, language, and executive functions, and are associated with academic underachievement and reduced school engagement (Lansing et al., 2004; Kolk et al., 2011; Yvon et al., 2018; Rivella and Viterbori, 2021; Abgottspon et al., 2022; Leung et al., 2023; Salzmann et al., 2025). The nature and severity of these outcomes are influenced by factors such as lesion location, age at onset, and the presence of comorbidities (Greenham et al., 2017a; Xu et al., 2022).

Beyond the visible consequences of stroke, there is growing recognition of the unseen struggles experienced by pediatric stroke survivors and their families. Emotional disorders, such as anxiety, depression, and post-traumatic stress symptoms, are frequently reported, yet often receive less attention than physical impairments (Greenham et al., 2017a,b, 2018; Badar et al., 2020; Lehman et al., 2020; Li et al., 2021). These emotional difficulties are frequently accompanied by social challenges, including problems in peer relationships, reduced participation in group settings, and deficits in social functioning (O'Keeffe et al., 2012; Anderson et al., 2014; Greenham et al., 2017a,b, 2018; Badar et al., 2020; Rivella et al., 2023a). Recent evidence suggests that these psychosocial difficulties can persist into adolescence and adulthood, potentially affecting educational achievement, employment opportunities, and quality of life (Elbers et al., 2014; Rohner et al., 2020; Hurvitz et al., 2004). Of note, these effects are not limited to the child. Stroke in a pediatric patient places substantial emotional and practical strain on the family system. Parents often report elevated levels of stress, anxiety, and depressive symptoms, which may in turn affect their capacity to provide emotional support and stable caregiving (Bemister et al., 2014, 2015; Labrell et al., 2018). At the same time, family cohesion, adaptive coping, and access to psychosocial resources have been identified as important protective factors (Labrell et al., 2018). Despite this, psychosocial outcomes remain underrepresented in pediatric stroke research, especially when compared to the emphasis placed on physical and cognitive sequelae, as well as to the broader adult stroke literature. Several interrelated factors likely account for this underrepresentation. The low incidence of pediatric stroke often results in research and clinical efforts being primarily directed toward immediate physical outcomes and survival, whereas psychosocial difficulties- frequently subtle, protracted, and challenging to quantify with standardized instruments- receive comparatively less systematic evaluation (Greenham et al., 2017a; Lo et al., 2014; Smithson et al., 2025). Moreover, conventional clinical protocols tend to prioritize neurological and motor assessments, with psychological and family functioning evaluations relegated to secondary importance (Bemister et al., 2014; O'Keeffe et al., 2012; Rivella et al., 2023b). The paucity of longitudinal and multicenter investigations documenting the persistence and impact of anxiety, depression, behavioral disturbances, and family dysfunction further contributes to a substantial knowledge gap, limiting the development and implementation of evidence-based psychosocial interventions (Bemister et al., 2015; Lehman et al., 2020; Smithson et al., 2025). As a result, many of these challenges remain insufficiently understood and inadequately addressed in clinical settings.

This narrative review aims to synthesize existing literature on the psychosocial and family outcomes associated with pediatric stroke. Specifically, it focuses on five key domains: (1) internalizing and externalizing disorders, including anxiety, depression, post-traumatic stress symptoms, and behavioral difficulties; (2) social-emotional functioning, including peer relationships and social participation; (3) the impact of pediatric stroke on the family system, including caregiver wellbeing and family dynamics; and (4) intervention strategies targeting psychosocial adjustment and family resilience, included to provide a clear link between the identification of psychosocial challenges and the implementation of evidence-based strategies to support affected children and families. This ensures that the review not only describes outcomes but also highlights practical approaches for clinical management and future research; (5) in addition to these core domains, health-related quality of life (HR-QoL) is considered as an integrative outcome, reflecting the cumulative impact of psychological symptoms, social functioning, and family dynamics, providing a comprehensive perspective on pediatric stroke survivors' overall wellbeing.

## 2 Method

### 2.1 Search strategy

This narrative review examines the psychological and psychosocial impact of pediatric stroke on affected children and their families, including psychosocial outcomes such as social functioning, peer relationships, and social participation. A narrative approach was selected due to the substantial heterogeneity of the available studies, which vary in design, participant characteristics, stroke subtypes, age at onset, follow-up duration, and outcome measures, including internalizing and externalizing symptoms, quality of life, social functioning, and family impact. The literature was systematically searched in PubMed and Web of Science. These databases were selected because, together, they provide comprehensive and complementary coverage of biomedical, neurological, and psychosocial research relevant to pediatric populations. Considering the substantial overlap in indexing with other major platforms, these two databases were deemed sufficient to capture the relevant peer-reviewed literature for the purposes of this narrative review. The search was conducted in November 2025.

A comprehensive search was performed using a combination of keywords related to pediatric stroke and psychosocial outcomes, including:

- Population: “pediatric stroke,” “childhood stroke”
- Internalizing outcomes: “internalizing symptoms,” “anxiety,” “depression,” “PTSD”
- Externalizing: “externalizing behaviors,” “conduct problems”
- Social outcomes: “social functioning,” “peer relationships”
- Quality of life: “quality of life”
- Family and caregiver outcomes: “caregiver stress,” “family dynamics”
- Interventions: “psychotherapy,” “psychological interventions”

An example of a search string used:

(“pediatric stroke” OR “childhood stroke”) AND (“internalizing symptoms” OR “anxiety” OR “depression” OR “PTSD” OR “externalizing behaviors” OR “social functioning” OR “quality of life” OR “caregiver stress” OR “family dynamics” OR “psychotherapy” OR “psychological interventions”).

### 2.2 Inclusion and exclusion criteria

Studies were eligible for inclusion if they involved children and adolescents (0–18 years) who had experienced a pediatric or neonatal stroke. We considered studies reporting psychological, behavioral, social, quality of life, or family-related outcomes. Eligible study designs included cross-sectional, longitudinal cohort, and case-control investigations. Only peer-reviewed publications in English published between 2000 and November 2025 were considered. Reference lists of retained papers were also examined to identify additional relevant studies. Studies were excluded if they involved exclusively adult populations (>18 years), focused solely on neurological or physical outcomes, or were non-peer-reviewed publications such as conference abstracts, editorials, or commentaries. Additionally, studies with very small sample sizes were excluded unless they provided unique clinical insights. For the purpose of this review, studies with fewer than 10 participants were considered to have a very small sample size, a threshold chosen to balance the inclusion of informative data in this rare population with the need to limit the influence of underpowered studies.

### 2.3 Study selection

The study selection process was conducted in two phases.

**Figure 1:**
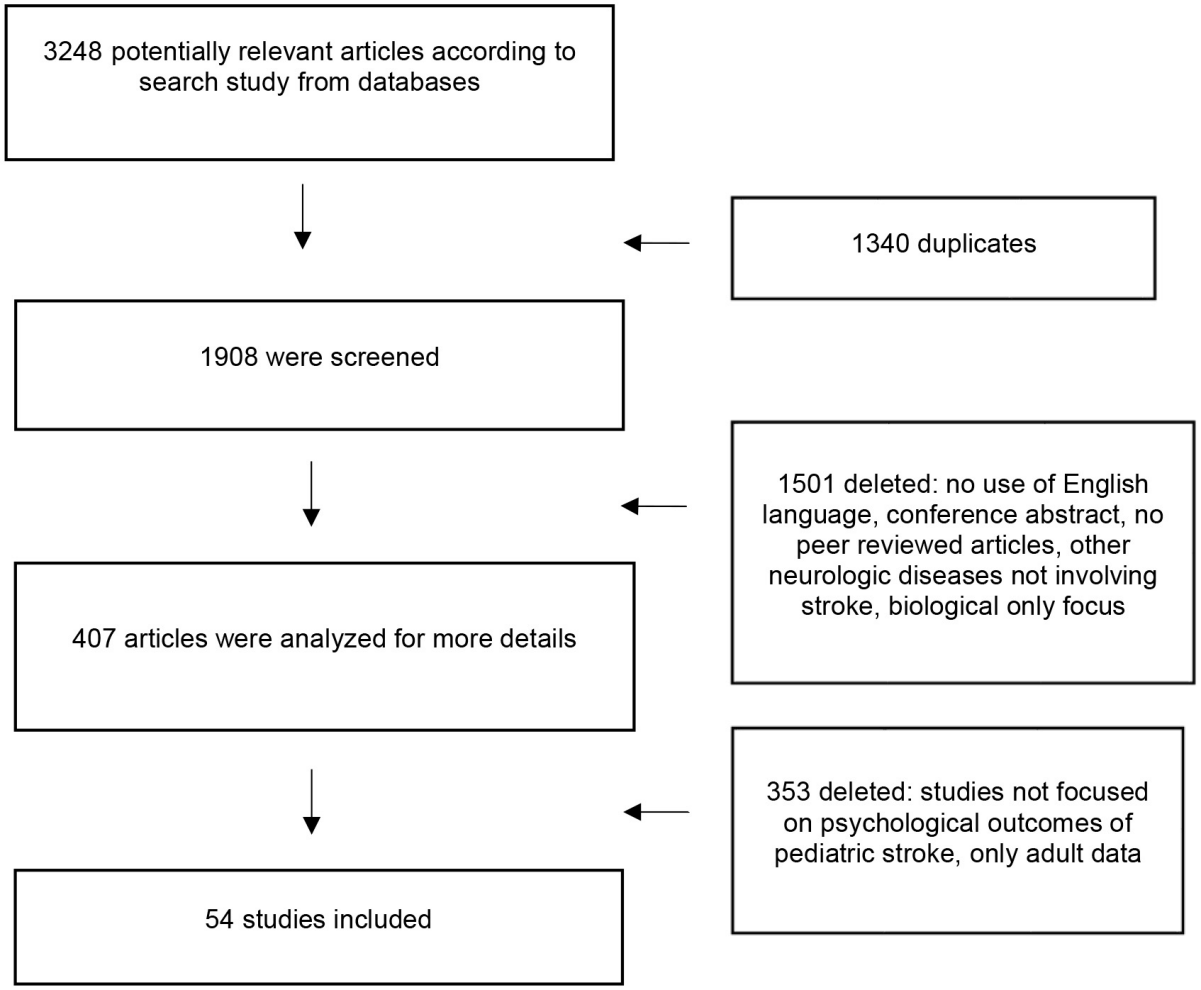
Flow diagram of the study methodology.

**Phase 1—Title and abstract screening:** During the initial screening phase, duplicate articles identified through database searches were removed to ensure that only unique records were considered. Two independent reviewers (S.T. and M.P.C.) then screened the titles, abstracts, and keywords of the remaining articles to assess their relevance to the review's focus on psychological, social, and family outcomes following pediatric stroke. Articles that clearly did not address these domains were excluded at this stage (Figure 1). In cases where relevance was ambiguous or the two reviewers disagreed, full-text versions of the articles were retrieved for a more detailed evaluation.

**Phase 2—Full-text screening:** Four additional reviewers (G.M., M.A.N.F., A.B., and G.T.) independently examined the full texts to determine whether the studies met the inclusion and exclusion criteria detailed in Section 2.2, ensuring that relevant psychological, social, or family outcomes were reported. Studies that did not meet these criteria were excluded. Any discrepancies were resolved by consensus under the supervision of the senior author (M.V.). Inter-rater agreement was assessed qualitatively: all disagreements between reviewers were discussed and resolved by consensus, with supervision from the senior author. As this was a narrative review, no formal statistical measure of inter-rater agreement was calculated. Main reasons for exclusion at the full-text stage were recorded and are presented in Figure 1.

### 2.4 Data extraction and synthesis

Data were extracted for each included study, including:

- Participant characteristics (age at stroke, stroke type, sample size, control group).
- Psychological and social outcomes (internalizing and externalizing symptoms, social functioning, HR-QoL, family-related factors).
- Evaluation methods and study procedures.

The synthesis is structured according to the key domains outlined in the introduction: internalizing and externalizing symptoms, social functioning, HR-QoL, family-related factors, and intervention strategies.

The narrative synthesis involved the following steps:

1. Preliminary synthesis: organizing studies according to thematic domains, including internalizing and externalizing symptoms, social functioning, HR-QoL, and family-related factors.
2. Exploring relationships: examining studies collectively to identify common trends, consistencies, discrepancies, and potential interactions between child psychological outcomes and family functioning.
3. Assessing the synthesis: key findings were described qualitatively, with studies organized according to outcome domains (e.g., internalizing and externalizing symptoms, social functioning, family-related factors). This approach allowed a clear overview of how clinical and contextual variables are associated with psychosocial and family outcomes in pediatric stroke.

A basic, qualitative consideration of study quality was applied. No formal scoring instrument was used, consistent with the narrative nature of this review. Study quality was taken into account narratively when synthesizing findings, with greater weight given to studies judged to be methodologically stronger.

## 3 Results

### 3.1 Search results and study selection

The comprehensive search identified 3,248 potentially relevant articles. After removing 1,340 duplicates, 1,908 unique records were screened by title and abstract. During this stage, 1,501 articles were excluded for reasons such as non-English language, conference abstracts or other non-peer-reviewed publications, focus on conditions other than stroke, or reporting only biological or physical outcomes. An additional 353 articles were excluded because they did not assess psychological, social, or family outcomes, or included only adult participants (Figure 1). A total of 54 studies met the inclusion criteria, evaluating psychological, social, or family outcomes following pediatric stroke. Samples varied widely in age at assessment (from infancy to late adolescence) and time since stroke (a few months to approximately 20 years), with sample sizes ranging from 10 to over 100 participants. Most studies were observational, with cross-sectional or longitudinal designs, while several included comparative or case-control elements (e.g., Anderson et al., 2014; Bemister et al., 2014). One study used a quantitative and qualitative design based on semi-structured parent interviews (Leal Martins et al., 2021). No randomized controlled trials were identified. Outcomes were assessed using standardized tools capturing quality of life, social functioning (e.g., peer relationships, social participation), psychological functioning (e.g., anxiety, depression, internalizing/externalizing symptoms), and parental or family wellbeing (e.g., caregiver stress, family dynamics). Detailed study characteristics, including sample, assessment instruments, and key findings, are summarized in Table 1.

**Table 1:** Summary of articles included in the review.

References	Sample description	Participants age	Stroke age/time since stroke	Measures	Findings
Anderson et al. (2014)	*N* = 36 children with AIS *N* = 15 children with asthma (medical control) *N* = 43 healthy control	AIS mean age: 9.1 ± 3.0 years (range: 6–15 years) Asthma group: 9.8 ± 2.6 years Control: 10.3 ± 3.0 years	Age at stroke (mean) 4.2 ± 4.3 years Time since stroke (mean) 5.0 ± 3.3 years	- CBCL (Child Behavior Checklist, parent report) - CASP (Child and Adolescent Scale of Participation, parent report) - FQQ (Friendship Quality Questionnaire, child report) - NRI (Network of Relationships Inventory, child report) - RPQ (Revised Peer Experiences Questionnaire, child report) - ABAS-II Social Subscale (Adaptive Behavior Assessment System, Second Edition-Social Subscale, parent report) - Harter Self-Perception Profile-Social Competence Scale (child report)	Children with arterial ischemic stroke demonstrated reduced social participation and increased internalizing problems compared to both control groups. However, self-reported social interaction did not differ significantly between groups. Key predictors of these outcomes included subcortical lesion location, the extent of neurological impairment, and lower levels of family functioning
Bemister et al. (2014)	*N* = 82 mothers of children with perinatal AIS (mild or moderate/severe) *N* = 62 mothers of healthy children *N* = 28 mother-father couples of AIS patients	Children with stroke (mean): 7.34 ± 5.20 years Control: 7.49 ± 5.15 years Parents stroke (mean): 38.05 ± 6.64 years Parents control: 37.82 ± 7.23 years	Age at stroke ≤ 28 days old	- HADS (Hospital Anxiety and Depression Scale) - PSS (Perceived Stress Scale) - PedsQL FIM (Pediatric Quality of Life Inventory- Family Impact Module) - POM (APSP Parental Outcome Measure) - DAS (Dyadic Adjustment Scale) - KMSS (Kansas Marital Satisfaction Scale)	Moderate/severe group had higher depression, stress, lower quality of life & marital satisfaction. Mothers reported more anxiety and feelings of guilt than fathers
Bemister et al. (2015)	*N* = 103 parents (76 mothers, 27 fathers) of children with confirmed perinatal AIS No control	Child age (age): 7.46 ± 5.42 (range: 0.5–18) Parent age (mean): 39.26 ± 1.70 (range: 26–59)	Time since stroke (mean) 5.59 ± 4.58 (range: 0.5–18) Time since first clinical presentation (mean) 6.41 ± 5 (range: 1–18 years)	- HADS (Hospital Anxiety and Depression Scale) - PedsQL FIM (Pediatric Quality of Life Inventory-Family Impact Module) - PSS (Perceived Stress Scale) - DAS (Dyadic Adjustment Scale) - POM (APSP Parental Outcome Measure) - DAS (Dyadic Adjustment Scale) - KMSS (Kansas Marital Satisfaction Scale)	Condition severity, anxiety, stress, and perceived blame emerged as significant predictors of caregiver depression. Similarly, condition severity, marital quality, and stress were significant contributors to family functioning. Blame was identified as a mediator in the relationship between condition severity and caregiver depression. No significant moderators or mediators were observed in the models predicting family functioning
Blom et al. (2003)	*N* = 56 patients with HS No control	Mean age: 18.6 years (range: 1.8–34.1 years)	Age at stroke (mean) 7.7 years (range: 1 month−15.9 years) Time since stroke (mean) 10.3 years (range: 1.3–19.9 years)	- CHQ-PF (Child Health Questionnaire-Parent Form, parent report) - CHQ-CF (Child Health Questionnaire- Child Form, child report) - SF-36 (36-Item Short Form Survey, self-report, for >15 years) A series of neuropsychological tests	Children reported lower self-esteem and more limitations in family activities. Parents reported more emotional and behavioral problems, including anxiety, depression, and school/social limitations. Parents also experienced significant emotional distress. Quality of life scores in older participants were slightly lower in physical domains compared to controls, though the difference was not statistically significant
Bulder et al. (2011)	*N* = 40 children with stroke No control	Median age: 9.2 years (range: 1.8–19.8 years)	Age at stroke (median) 5.6 years (range: 0.3–15.9 years) Time since stroke (median) 3.1 years (range: 0.6–13.1 years)	- PSOM (Pediatric Stroke Outcome Measure, clinician-rated) - mRS (modified Rankin Scale, clinician-rated) - PedsQL (Pediatric Quality of Life Inventory, child and parent report) - VAS (Visual Analog Scale, child and parent report)	The majority of children experienced lasting neurological deficits, and a notable proportion required special education services. Children with more favorable neurological outcomes reported higher quality of life. Overall, the severity of neurological impairment emerged as the strongest predictor of quality of life
Christerson and Strömberg (2010)	*N* = 46 children (22 AIS, 20 HS and 4 SVT; *n* = 23 < 18) No control	Median age: 17.5 years (range: 5.5–26.1 years)	Time since stroke (median) 4.2 years (range: 1.6–8.6 years)	- CHQ-CF (Child Health Questionnaire—Child Form) - CHQ-PF (Child Health Questionnaire—Parent Form)	Parents reported limitations in their children's social interactions, attributed to emotional difficulties. However, no significant differences in internalizing symptoms were observed in the reported data
Cnossen et al. (2010)	*N* = 76 children with AIS No control	Median age: 2.5 years (range: 1 month−17.2 years)	Age at stroke (median) 2 years and 5 months (range: 1 month−17.2 years) Time since stroke (median) 2 years and 4 months (range: 7 months−10.6 years)	- TAPQOL (TNO-AZL Preschool Children Quality of Life Questionnaire, parent report) - TACQOL-PF (TNO-AZL Child Quality of Life Questionnaire-Parent Form, parent report) - TACQOL-CF (TNO-AZL Child Quality of Life Questionnaire-Child Form, child report) - TAAQOL (TNO-AZL Adult Quality of Life Questionnaire, child report)	Increased anxiety, less positive mood, and higher emotional and social problems reported in children post-stroke
De Schryver et al. (2000)	*N* = 37 children with AIS at baseline *N* = 27 at follow-up No control	Mean age: 11.6 years (range: 3–25 years)	Age at stroke (mean) 4.6 years (range: 3 months−14 years) Time since stroke (mean) 7.1 years (range: 3 months−20 years years)	Unstandardized questionnaire (parent and child reports)	A substantial proportion of parents reported changes in their child's behavior within the school environment and a reduction in peer relationships. Developmental stagnation was also commonly observed. Nonetheless, the majority of parents perceived their child as experiencing a level of happiness comparable to that of their peers
Everts et al. (2008)	*N* = 21 children with AIS No control	Mean age: 11.1 ± 4.3 years	Age at stroke (mean) 7.3 ± 4.6 years (range: 1 month−17.6 years) Time since stroke (mean) 4.9 ± 3.10 years (range: 14 days−14 years)	- Conners' Parent Rating Scales (parent report) - KIDSCREEN-52 (child and parent report)	Children with AIS were reported to experience higher levels of anxiety, greater shyness, more emotional difficulties, and lower perceived social acceptance
Friefeld et al. (2004)	*N* = 100 children (84 AIS, 16 CSVT) No control	Mean age: 8.4 ± 4.12 years	Age at stroke 0–18 years Time since stroke (mean) 4.4 ± 2.93 years	- PedsQL 4.0 (Pediatric Quality of Life Inventory, child and parent report)	Stroke group had higher social and emotional difficulties; girls showed lower emotional function; poorer neurological outcome predicted more difficulties
Ghotra et al. (2015)	*N* = 90 children with AIS (31 perinatal, 36 neonatal, 23 childhood onset) No control	Perinatal (mean): 5 years and 2 months ± 3.4 (range: 2 years and 1 month−15 years and 2 months) Neonatal: 5 years and 2 months ± 3 (range: 2 years and 5 months−15 years and 1 month) Childhood: 9 years and 10 months ± 5.8 (range: 2 years and 2 months−17 years and 10 months)	Age at stroke (mean) Perinatal: ND Neonatal: 2 days ± 4 (range: 0–21) days Childhood: 5 years and 2 months ± 4.9 (range: 2 months−14 years) Time since stroke (mean) Perinatal: 5 years and 2 months ± 3.4 (range: 2 years and 1 month−15 years and 2 months) Neonatal: 5 years and 2 months ±3 (range: 2 years and 5 months−15 years and 1 month) Childhood: 4 years and 7 months ± 2.9 (range: 1 year−10 years and 8 months)	- PedsQL 4.0 (Pediatric Quality of Life Inventory, parent)	Children with AIS show lower social, emotional, and psychological functioning than norms. Age at stroke and lesion size predict outcomes. Perinatal stroke leads to the poorest clinical and functional outcomes and lowest independence. Neonatal stroke has the best quality of life, differing from norms only emotionally. Perinatal and childhood strokes reduce quality of life, with childhood stroke scoring lowest overall
Ghotra et al. (2018)	*N* = 59 children with AIS (36 neonatal onset, 23 childhood onset) No control	Mean age: 7 years (range: 2.2–17.9 years)	Age at stroke (mean) 2.2 years (range: 0–4 years) Age since stroke 5 years (range: 1–15.1 years)	- PedsQL 4.0 (Pediatric Quality of Life Inventory, child and parent report)	Parents reported more emotional difficulties than their children; however, both groups indicated reduced overall wellbeing. Health-related quality of life was predicted by neurological outcomes, stroke location, and socioeconomic status
Gordon et al. (2002)	*N* = 17 children with AIS No control	Median age: 8 years and 5 months (range: 5 years and 2 months−15 years and 5 months)	Age at stroke (median) 4 years (14 months−13.6 years) Time since stroke (media) 2 years and 5 months (10 months−8 years and 6 months)	- CHQ-50 (Child Health Questionnaire50 item, parent report)	Children with AIS show significant impairments in cognitive and sensorimotor functions. Their physical and psychosocial quality of life is much lower than typical peers. Communication difficulties strongly predict poorer psychological health. Parents also report reduced social and psychological functioning. Factors like age at stroke and emotional skills influence outcomes
Greenham et al. (2017a)	*N* = 31 children with AIS (13 neonatal onset, 18 childhood onset) No control	Neonatal group (mean): 5.1 ± 0.8 years (range: 3.8–6.5 years) Childhood group: 9.5 ± 3.0 years (range: 5.9–15.4 years)	Age at stroke Neonatal group: 0 years Childhood group (mean): 4.2 ± 2.8 years Time since stroke Neonatal group (mean): 5.1 ± 0.8 years Childhood group: 5.3 ± 0.8 years	- SDQ (Strengths and Difficulties Questionnaire, parent report) - VABS-II (Vineland Adaptive Behavior Scales- Socialization Subscale, clinician/parent report) - SSIS (Social Skills Subscale, parent report)	Children who experienced a stroke exhibited elevated emotional symptoms and reduced prosocial behavior compared to normative data. Poor cognitive and psychological functioning at 12 months post-stroke, older age at stroke onset, and larger lesion size were significant predictors of poorer social outcomes
Greenham et al. (2017b)	*N* = 31 patients with AIS (13 neonatal, 18 childhood onset) No control	Neonatal group (mean): 5.1 ± 0.8 years (range: 3.8–6.5 years) Childhood group: 9.5 ± 3.0 years (range: 5.9–15.4 years)	Age at stroke Neonatal group: 0 years Childhood group (mean): 4.2 ± 2.8 years Time since stroke Neonatal group (mean): 5.1 ± 0.8 years Childhood group: 5.3 ± 0.8 years	- VABS-II (Vineland Adaptive Behavior Scales-Socialization Scale, clinician/parent report) - SDQ (Strengths and Difficulties Questionnaire, parent report)	Social impairment was found to increase over time and was significantly associated with psychological difficulties. Poor socialization outcomes were predicted by older age at stroke onset and the presence of neurological impairments
Kornfeld et al. (2017)	*N* = 70 children with AIS (excluded pre-stroke developmental delay) No control	Median age: 13.5 years (range: 6.25–19 years)	Age at stroke 3 months−15 years and 7 months (median 8.25 years) Time since stroke 5 years	- Kidscreen-27 (Kidscreen-27 Quality of Life Questionnaire) - DSM-IV ADHD Screening (Diagnostic and Statistical Manual of Mental Disorders, Fourth Edition-ADHD screening) - ABILHAND-Kids (Manual Ability Measure for Children with Cerebral Palsy)	Psychological wellbeing was generally good and often above average. Physical health, autonomy, and school experience were rated positively, while social support was consistently low. Lower intellectual functioning and attention difficulties were associated with reduced psychological wellbeing. Physical and motor impairments also correlated with lower quality of life. The combination of neurological and cognitive difficulties predicted poorer mental health outcomes
Labrell et al. (2018)	*N* = 78 mothers of children with brain tumors (*N* = 37) or other ABI (*N* = 41)	Children (mean): 9.3 ± 5.1 years Mothers: 39.2 ± 6.4 anni	Age at diagnosis (mean) 7.7 ± 5.0 anni Time since diagnosis (mean) 1.5 ± 2.2 anni	- PIP (Pediatric Inventory for Parents) - STAI (State-Trait Anxiety Inventory) - FAD (Family Assessment Device)	No significant differences were found between the tumor and other acquired brain injury groups in levels of stress, anxiety, or family functioning. Parental stress was higher compared to families of children with inflammatory bowel disease and non-brain cancers. A substantial proportion of mothers reported elevated state anxiety. Overall, family functioning was generally reported as good
Leal Martins et al. (2021)	*N* = 14 neonates with NAIS (qualitative part included 9 infants' families)	Neonates (birth to first days of life)	Age at stroke within first 2 days of life (mean 27 h after birth) Time since stroke 2–10 years	Semi-structured parent interviews	Despite generally good child quality of life, parents reported ongoing anxiety. Qualitative findings revealed delayed symptom recognition, shock at diagnosis, and persistent concerns about development. Mild motor or attention issues in some children contributed to stress, impacting overall family quality of life
Ledochowski et al. (2020)	*N* = 75 children with AIS (24 with dystonia, 51 without dystonia) No control	Mean age: 11.9 ± 3.3 years	Age at stroke (mean) ± 4.2 years Time since stroke (mean) 8.4 ± 4.5 years	- BASC-2 PRS (Behavior Assessment System for Children, Second Edition-Parent Rating Scales)	Children with AIS demonstrated a heightened risk of internalizing problems, with this risk further exacerbated by the presence of dystonia. Those with dystonia showed elevated levels of anxiety and depression, with emotional symptoms appearing largely independent of motor and cognitive functioning. In contrast, in children without dystonia, motor and cognitive outcomes were significantly correlated
Lehman et al. (2020)	*N* (whole sample) = 57 children with stroke (AIS or HS) *N* = 81 parents (54 mothers, 27 fathers) No control	Children age range: 7–18 years Parents of children 0–18 years	Age at stroke: neonatal to adolescence Assessment T1: < 2 years post-stroke; T2: 6 months follow-up	- BAI (Beck Anxiety Inventory) - BDI-II (Beck Depression Inventory-II) - PCL-5 (PTSD Checklist for DSM-5) - RRQ (Rumination-Reflection Questionnaire) - BASC-2 (Behavior Assessment System for Children-Second Edition) - UCLA PTSD RI (UCLA Posttraumatic Stress Disorder Reaction Index)	Mothers reported elevated symptoms of post-traumatic stress and depression, which were associated with greater perceived disability in their child. These symptoms tended to improve over time. While some children reported symptoms of anxiety and depression, these were not consistently associated with parental psychological outcomes. Stroke type and medical complications did not demonstrate a significant impact on emotional outcomes
Lo et al. (2014)	*N* = 36 children with AIS (10 perinatal, 26 childhood onset) *N* = 15 children with asthma (comparison group)	Median age: 8.5 years (range: 6.5–11.8 years)	Time at stroke (median) 2.6 years (range: 0–7.8 years)	- CBCL (Child Behavior Checklist, parent report) - ABAS-II Social Subscale (Adaptive Behavior Assessment System-Social Subscale, parent report) - CASP (Child and Adolescent Scale of Participation, parent report)	No significant differences in internalizing problems were found between children with AIS and those with asthma. However, children with AIS exhibited greater difficulties in peer relationships and lower levels of social participation. Poor neurological functioning and lower intellectual ability were significant predictors of poorer social adjustment
Lo et al. (2020)	*N* = 10 children with stroke *N* = 10 healthy control	Patients median age: 12.5 years (range: 8–17 years) Control median age: 13.0 years (13–17 years)	Time since stroke (median) 5.2 years (range: 0–17 years)	- Irony and Empathy Task - Emotional and Emotive Faces Task - CASP (Child and Adolescent Scale of Participation, parent report) - ABAS-II (Adaptive Behavior Assessment System, Second Edition, parent report)	Children with stroke demonstrated poorer performance on Theory of Mind tasks and engaged less in social activities compared to their typically developing peers
Max et al. (2002)	*N* = 29 children with stroke (AIS or HS): 17 early-onset, 12 late-onset *N* = 29 orthopedic controls (clubfoot or scoliosis)	Early-onset stroke: 11.8 years ± 3.6 Late-onset stroke: 13.2 years ± 4.2 Controls: age-matched	Age at stroke Early-onset: < 12 months Late-onset: ≥12 months Time since stroke ≥1 year post-stroke	- K-SADS-PL (Kiddie Schedule for Affective Disorders and Schizophrenia-Present and Lifetime Version, clinician/parent report) - VABS-SS (Vineland Adaptive Behavior Scales- Socialization Subscale, clinician/parent report)	Pediatric stroke was associated with an increased risk of internalizing psychiatric symptoms and diminished psychosocial functioning compared to control subjects. These outcomes were significantly predicted by the severity of neurological impairment and the anatomical location of the cerebral lesion
O'Keeffe et al. (2012)	*N* = 49 children with AIS No control	Mean age: 11.08 ± 3.65 years	Age at stroke (mean) 5.08 ± 3.67 years Time since stroke (mean) 6.0 ± 3.41 years	- PedsQL (Pediatric Quality of Life Inventory) - CFSEI III (Cultural Fair Intelligence Test, Third Edition) - SES (Socioeconomic Status) - GHQ-12 (General Health Questionnaire, 12-item version) - WASI (Wechsler Abbreviated Scale of Intelligence) - WIAT-II (Wechsler Individual Achievement Test, Second Edition) - WOLD (Woodcock Oral Language Development Test) - TEA-Ch (Test of Everyday Attention for Children) - D-KEFS (Delis-Kaplan Executive Function System) - BRIEF (Behavior Rating Inventory of Executive Function) - SDQ (Strengths and Difficulties Questionnaire)	Health-related quality of life reported by both children and parents was lower than normative values across all domains. There was a greater negative impact on family functioning and parental wellbeing. Children with individualized education plans were more severely affected
Peterson et al. (2021)	*N* = 30 parents (mostly mothers) of children with neonatal AIS	Mean child age: 3.70 ± 1.71 years Mean parent age: 34.00 ± 5.28 years	Age at stroke ≤ 28 days old Time since stroke 3 years	- PEQ (Parent Experiences Questionnaire) - DASS (Depression Anxiety Stress Scale)—CBCL (Child Behavior Checklist) - Bayley Scales of Infant Development—Third Edition - WPPSI-IV (Wechsler Preschool and Primary Scale of Intelligence-Fourth Edition) - WISC-V (Wechsler Intelligence Scale for Children-Fifth Edition) - PSOM (Pediatric Stroke Outcome Measure)	Children showed no significant differences from typical norms in overall cognition and language, though some experienced notable difficulties and elevated externalizing behaviors. Parents generally had average mental health, but with wide variability; higher parental stress, anxiety, and depression were associated with more externalizing symptoms in children, and parental depression was linked to lower cognitive scores. Parents mainly recalled discussions focusing on motor, cognitive, and language outcomes, with less emphasis on social, behavioral, or mental health aspects
Rivella et al. (2023b)	*N* = 30 children with perinatal or childhood AIS No control	Mean age: 9.44 ± 1.65 years (range: 7–12 years old)	Age at stroke (mean) 4.93 ± 3.84 years Time since stroke (mean) 4.59 ± 3.38 years	- CPM (Colored Progressive Matrices) - Numerical Stroop (Numerical Stroop Task) - NEPSY-II inhibition (NEPSY-II Inhibition Subtest) - Listening Span (Listening Span Task) - Backward Digit Span (Backward Digit Span Task) - Trail Making Test (Trail Making Test) - WISC-IV Cancellation (Wechsler Intelligence Scale for Children-Fourth Edition, Cancellation Subtest) - SDAG (Strengths and Difficulties Attention-Deficit Group Scale) - SDQ (Strengths and Difficulties Questionnaire) - PSOM (Pediatric Stroke Outcome Measure)	Children with executive function difficulties were reported by their parents to exhibit increased emotional symptoms, attentional problems, elevated hyperactivity, and difficulties in peer relationships. A substantial proportion of these children experienced emotional difficulties reaching clinically significant levels. Executive function deficits were associated with an increased risk of psychosocial impairment
Ryan et al. (2021)	*N* = 30 children with AIS (12 neonatal) *N* = 37 healthy control	AIS mean age: 7.85 ± 3.12 years Controls: 7.80 ± 2.79 years	Age at stroke 1.45 years (IQR 4.04) Time since stroke 5.26 ± 0.76 years	- NEPSY-II (Neuropsychological Assessment, Second Edition- emotion recognition, cognitive and affective Theory of Mind) - SDQ (Strengths and Difficulties Questionnaire) - PSOM (Pediatric Stroke Outcome Measure)	Children in the arterial ischemic stroke group performed significantly worse than controls in emotion recognition, cognitive theory of mind, affective theory of mind, and overall theory of mind abilities. Poorer outcomes were associated with larger lesions, involvement of both cortical and subcortical regions, and infarcts affecting multiple vascular territories. Lower performance in cognitive theory of mind was linked to increased peer relationship difficulties and reduced prosocial behavior
Smith et al. (2015)	*N* = 59 children with AIS and HS (21 perinatal) No control	Median age: 10.5 years (8.4–14.5 years)	Age at stroke (median) 11 years and 6 months (6 years and 4 months−13 years and 5 months) Time since stroke (median) 3 years and 9 months (1 years and 7 months−8 years and 6 months)	- PedsQL (Pediatric Quality of Life Inventory and Cerebral Palsy Module; child and parent report)	Lower health-related quality of life than norms; psychosocial health scores lower than physical; hemiparesis, epilepsy, and lower IQ associated with poorer outcomes
Smithson et al. (2025)	*N* = 77 children with perinatal AIS (APPIS, NAIS, or PVI) No control	Mean age: 9.3 ± 3.3 years	Age at stroke Perinatal Time since stroke about 9 years (range: 4–18 years)	- BASC-3 (Behavior Assessment System for Children, Third Edition) - PedsQL (Pediatric Quality of Life Inventory) - PSQ-SRBD (Pediatric Sleep Questionnaire-Sleep-Related Breathing Disorder scale) - POM (Parental Outcome Measure, parent report)	Children had elevated externalizing, internalizing, and behavior symptom scores and lower adaptive skills compared to norms. Sleep-disordered breathing associated with worse mental health, quality of life, and greater caregiver stress. Motor and epilepsy comorbidities linked to poorer quality of life

AIS, Arterial Ischemic Stroke; HS, Hemorrhagic Stroke; CSVT, Cerebral Sinovenous Thrombosis; NAIS, Neonatal Arterial Ischemic Stroke; APPIS, Apparent Perinatal Ischemic Stroke; PVI, Periventricular Venous Infarction; ABI, Acquired Brain Injury.

### 3.2 Internalizing symptoms: anxiety, depression, and PTSD

#### 3.2.1 Prevalence and patterns

Internalizing disorders, such as anxiety, depression, and post-traumatic stress symptoms, are common psychological sequelae following pediatric stroke (Greenham et al., 2017b, 2018; Lehman et al., 2020; Rivella et al., 2023a). Prevalence rates vary widely, with anxiety ranging from 4% to 36% and depression from 4% to 25%, reflecting heterogeneity in methodology and clinical populations (Max et al., 2002; Blom et al., 2003; Everts et al., 2008; Williams et al., 2017, 2019; Westmacott et al., 2018; Ledochowski et al., 2020; Table 1). In an early study, Max et al. (2002) reported significantly higher rates of anxiety (31%), mood disorders (21%), and ADHD (46%) in pediatric stroke survivors compared to controls. Although psychological difficulties after pediatric stroke have been widely documented, some research has reported a lack of significant psychopathological symptoms in this population. (Christerson and Strömberg 2010) found that children with stroke reported higher self-esteem than healthy controls and did not perceive the same limitations in social interactions or overall health as those reported by their parents. In contrast, parents described health-related problems and increased caregiving demands. Although most children's scores were comparable to controls, some demonstrated reduced functioning in specific domains. Parent-child agreement was high for bodily pain and family cohesion but low for general health and social limitations, underscoring discrepancies between perceived and observed outcomes (Christerson and Strömberg, 2010). Similarly, Lo et al. (2014) reported no differences in internalizing symptoms between children with AIS and those with asthma. Discrepancies between parent and child reports of emotional and social difficulties are common. Blom et al. (2003) reported that while both children and parents acknowledged low self-esteem and some impact on activities, parents described more pronounced emotional and functional difficulties, including higher levels of anxiety and depression and greater limitations in schoolwork and peer relationships.

#### 3.2.2 Development and outcomes

Longitudinal research has provided insights into the developmental trajectory of emotional and behavioral outcomes following pediatric stroke. Greenham et al. (2017a,b) conducted longitudinal assessments at 6 months, 12 months, and 5 years post-stroke. During the first year, increases in emotional difficulties, particularly anxiety and inattention, were observed. At the 12-month follow-up, participants continued to exhibit elevated levels of hyperactivity and inattention compared to normative data; however, no statistically significant group-level differences were observed in emotional or conduct symptoms. At the 5-year follow-up, patients exhibited increased emotional symptoms, hyperactivity, and overall psychological difficulties (Greenham et al., 2017a). Subsequent research by Lehman et al. (2020) confirmed the persistence of clinically significant anxiety (24%), depression (14%), and PTSD (6%) 2 years post-stroke, highlighting the chronic nature of these emotional sequelae. More recently, Rivella et al. (2023b) reported that children aged 7–12 years with preserved intellectual abilities following AIS frequently exhibited emotional and behavioral difficulties.

#### 3.2.3 Clinical and developmental considerations

Evidence from the literature indicates that developmental timing and clinical characteristics are associated with psychological outcomes in children following stroke. In the above-mentioned study by Greenham et al. (2017a), poorer emotional and behavioral outcomes at the 5-year follow-up were associated with older age at stroke onset, with behavioral difficulties particularly pronounced among children who experienced stroke later in development. Moreover, early emotional and cognitive difficulties, together with greater lesion size, emerged as the strongest predictors of long-term psychological outcomes. In contrast, early neurological status and psychological functioning at the 12-month follow-up did not significantly predict 5-year outcomes. Conversely, early neurological status and psychological functioning at the 12-month follow-up were not significant predictors of 5-year outcomes. Neurological sequelae following stroke, particularly post-stroke dystonia, may further affect long-term outcomes. Ledochowski et al. (2020) reported that children who developed acquired dystonia after a basal ganglia stroke exhibited significantly higher anxiety and depressive symptoms compared to peers with similar strokes without dystonia. In the dystonia group, mental health outcomes were largely independent of motor and cognitive function, with the exception of a specific link between depressive symptoms and behavioral regulation difficulties.

### 3.3 Externalizing behaviors: hyperactivity, impulsivity, and behavioral dysregulation

Although less studied, externalizing behaviors such as hyperactivity, impulsivity, and irritability are also present. In 210 children with ischemic stroke, approximately 10% exhibited clinically significant hyperactivity, strongly associated with deficits in inhibitory control and cognitive flexibility (Wilson et al., 2024). No major behavioral differences emerged between perinatal and childhood stroke, though perinatal stroke survivors scored higher on the BRIEF-2 “Shift” subscale, indicating difficulties in cognitive flexibility and adapting to change. These findings are supported by a recent study showing that children with perinatal stroke exhibit significantly elevated externalizing behavior scores compared to normative populations, with 27.1% in the at-risk or clinical range. In addition to stroke-related factors, other contributors to externalizing behaviors have been identified. Higher sleep-disordered breathing symptomatology has been shown to correlate with increased hyperactivity, impulsivity, and irritability, independently of motor impairments and epilepsy (Smithson et al., 2025).

### 3.4 Social functioning and peer relationships

#### 3.4.1 General outcomes

Children who survive pediatric stroke often experience significant and persistent impairments in social functioning, which can negatively affect emotional wellbeing and increase the risk of social withdrawal and isolation (O'Keeffe et al., 2012; Anderson et al., 2014; Greenham et al., 2017a,b, 2018; Badar et al., 2020; Rivella et al., 2023a; Table 1). In a comparative study involving children with AIS, peers with asthma, and healthy controls, Anderson et al. reported that the stroke group exhibited significantly poorer social adjustment and reduced participation (Anderson et al., 2014). These children frequently perceive lower levels of peer acceptance and social inclusion, with studies reporting higher rates of perceived peer rejection and reduced social integration, as observed by parents, teachers, and the children themselves (Friefeld et al., 2004; Everts et al., 2008; O'Keeffe et al., 2012; Greenham et al., 2015; Camilleri et al., 2025). Greenham et al. (2015) differentiated between social competence (capacity for empathy, communication, cooperation) and socialization (actual engagement in peer relationships). Despite often preserved foundational social skills, children frequently encountered difficulties in effectively applying these abilities within everyday social contexts. This distinction highlights that while core social capacities may remain intact, translation into meaningful peer interactions is often impaired following brain injury. Emerging evidence suggests that social difficulties are partly driven by deficits in social cognition, particularly in emotion recognition, empathy, and theory of mind (ToM), which contribute to reduced participation in peer activities and hinder effective application of social skills (Ryan et al., 2021; Lo et al., 2020). Despite these challenges, social difficulties can be perceived differently by children and adults: parents and teachers report more issues than children themselves, who may experience social rejection not fully recognized (Anderson et al., 2014; Greenham et al., 2015; Ghotra et al., 2018; Rivella et al., 2023a).

#### 3.4.2 Clinical and developmental considerations

Stroke-related variables, including lesion location, lesion volume, and age at onset, may interact with neurocognitive vulnerabilities to influence social outcomes. Children with AIS, as reported by Anderson et al., exhibited significantly poorer social adjustment and reduced participation compared to peers with asthma and healthy controls (Anderson et al., 2014). Subcortical lesion location and earlier age at stroke onset were associated with more favorable social outcomes and higher self-esteem. Greenham et al. (2017a,b) reported significant declines in socialization within the first year following pediatric stroke, with only partial and non-significant improvement at 12 months. At 5-year follow-up, impairments in socialization, measured by the Vineland Adaptive Behavior Scales-II (VABS-II) and the Social Skills Improvement System (SSIS), remained evident. Poorer social outcomes were significantly associated with older age at stroke onset. A substantial proportion of children exhibited clinically significant socialization difficulties, particularly those with comorbid motor or attentional impairments, highlighting the contribution of neurocognitive vulnerabilities. Even children with preserved cognitive abilities may show reduced social participation if environmental support is limited. Parental mental health and family functioning are strong predictors of children's social outcomes, including internalizing behaviors, social competence, and participation. Parental education also affects social competence, likely reflecting premorbid factors rather than the stroke itself (Greenham et al., 2015).

### 3.5 Health-related quality of life

#### 3.5.1 General outcomes

Beyond the acute and subacute consequences of pediatric stroke, the long-term impact on children's quality of life (QoL) is increasingly recognized as a complex and multidimensional outcome. While clinical recovery often focuses on neurological or motor status, QoL encompasses a broader range of experiences, including physical autonomy, emotional resilience, social belonging, and overall wellbeing, that may remain affected years after the stroke. Children who have experienced AIS frequently report lower QoL compared to healthy peers, particularly in domains such as emotional functioning, school participation, and peer relationships (Friefeld et al., 2004, 2011; Neuner et al., 2011; O'Keeffe et al., 2012; Akif-Özdemir et al., 2023; Fong et al., 2025; Table 1). Children classified as having a “good” outcome consistently demonstrate higher QoL ratings than those with “poor” outcomes, highlighting the influence of clinical recovery on subjective quality of life (Bulder et al., 2011). Children with neurological impairments may still report surprisingly high levels of subjective wellbeing, consistent with the “disability paradox” (Albrecht and Devlieger, 1999; Dickinson et al., 2007; Goeggel Simonetti et al., 2015). In a 2000 study, De Schryver et al. reported that approximately 75% of children perceived their health status as comparable to peers despite motor and cognitive challenges, with nearly all children expressing happiness and general satisfaction. Friefeld et al. (2011) found that nearly one-third of parents rated their child's QoL as “excellent,” reflecting resilience among pediatric stroke survivors.

#### 3.5.2 Clinical and developmental considerations

Neurological severity, motor impairment, cognitive deficits, epilepsy, and age at stroke onset have been consistently associated with differences in health-related quality of life (HR-QoL) among pediatric stroke survivors. The severity of neurological and motor outcomes is a well-established determinant of QoL across neurological populations (Smith et al., 2015; O'Keeffe et al., 2017; Caspar-Teuscher et al., 2019; Rivella et al., 2023a). Children with more severe disability (modified Rankin Scale, mRS = 3) tend to report lower scores in motor, cognitive, and autonomy domains compared to those with minimal or no disability (mRS = 1; O'Keeffe et al., 2017). Friefeld et al. (2004) identified neurological severity, measured by the Pediatric Stroke Outcome Measure (PSOM), as the strongest predictor of parent-reported HR-QoL, accounting for 16% of the variance. For child-reported HR-QoL, both neurological severity and sex were significant predictors, with girls reporting lower QoL than boys, particularly in emotional functioning and overall wellbeing. Children with moderate to severe motor impairments, especially in manual dexterity, scored up to 30% lower in the physical and social domains of HR-QoL compared to those with milder deficits (Friefeld et al., 2004). In addition to motor outcomes, other clinical factors significantly influence QoL and adaptive behavior (Max et al., 2002; Bulder et al., 2011; Friefeld et al., 2011; Lo et al., 2014; Smith et al., 2015). Age at stroke onset has also been associated with differences in long-term outcomes, with children who sustained a stroke later in childhood exhibiting better motor outcomes and higher HR-QoL scores compared to those with neonatal or early infancy strokes (Cnossen et al., 2010; Ghotra et al., 2015; O'Keeffe et al., 2017). Ghotra et al. reported that children who sustained a stroke later in childhood, compared to those with stroke in infancy, exhibited better motor outcomes and higher HR-QoL scores (Ghotra et al., 2015). These findings indicate an association between the developmental phase in which stroke occurs and subsequent functional and quality of life outcomes. Among post-stroke clinical conditions affecting recovery and quality of life, epilepsy, cognitive deficits, and motor functions represent significant contributors, with epilepsy and hemiplegia independently associated with lower parent-reported HR-QoL, particularly in physical and emotional domains (Smith et al., 2015). Cognitive impairments, particularly in verbal and executive functioning, negatively affect social functioning and overall wellbeing (Friefeld et al., 2011; O'Keeffe et al., 2012; Smith et al., 2015; Kornfeld et al., 2017). Lower verbal intelligence is linked to poorer social functioning and overall wellbeing, as reduced intellectual abilities often hinder communication, emotional regulation, and peer interactions (Friefeld et al., 2011; O'Keeffe et al., 2012; Smith et al., 2015). Moreover, executive dysfunction, often linked to greater disease severity, is associated with diminished HR-QoL due to its impact on emotional control, behavior, and academic performance, particularly in early-onset stroke or comorbid epilepsy, and is often intensified by family stress (De Schryver et al., 2000; Gordon et al., 2002; O'Keeffe et al., 2012, 2017; Greenham et al., 2015; Smith et al., 2015).

### 3.6 Parental psychological distress and family functioning following pediatric stroke

#### 3.6.1 General outcomes

Beyond the child's psychological adjustment, pediatric stroke exerts a significant emotional impact on parents, often manifesting in elevated levels of stress, anxiety, depression, and PTSD, especially among mothers (Bemister et al., 2014; Lehman et al., 2020; Peterson et al., 2021; Table 1). Lehman et al. (2020) assessed 81 parents of 57 children post-stroke and found that 28% of mothers and 15% of fathers exhibited clinical-range PTSD symptoms within 2 years after the stroke event (T1). Depression affected over a quarter of mothers and fathers, while clinical anxiety was less prevalent. Parental PTSD and depression were closely correlated, particularly in fathers, and maternal emotional distress was linked to a poorer perception of the child's recovery and functioning. Longitudinal follow-up at 6 months showed improvement in maternal PTSD and depression symptoms, though some parents developed new symptoms over time (Lehman et al., 2020). In a complementary study, Peterson et al. (2021) found that higher levels of parental anxiety, depression, and stress were associated with increased externalizing behaviors in children with neonatal stroke, suggesting that caregiver mental health may influence, and be influenced by, the child's behavioral adjustment.

#### 3.6.2 Factors influencing parental distress and family functioning

Parental psychological distress following pediatric stroke is shaped by multiple interrelated factors, including both child-related clinical characteristics and broader psychosocial processes. Bemister et al. examined the psychosocial consequences of perinatal stroke on caregivers, with particular attention to psychological distress, feelings of guilt, and marital dynamics (Bemister et al., 2014, 2015). In their 2014 study, mothers of children with moderate to severe outcomes reported significantly higher levels of depressive symptoms, reduced QoL, poorer family functioning, and lower marital satisfaction compared to mothers of typically developing children. Mothers also reported greater feelings of guilt and anxiety than fathers. The 2015 follow-up study demonstrated that although the severity of the child's condition was a relevant predictor of caregiver depression, psychosocial variables such as anxiety, stress, social support, and guilt were stronger and more consistent predictors. Parental feelings of guilt specifically mediated the relationship between neurological severity and depressive symptoms, highlighting the potential benefit of interventions targeting these modifiable psychosocial risk factors.

These individual psychological challenges often extend beyond the parent, influencing broader family dynamics. Family functioning and QoL are frequently compromised, with persistent difficulties in daily living reported by both children and their parents, particularly in self-care, productivity, and leisure (Galvin et al., 2010). Psychosocial factors, particularly marital quality and parental stress, often account for variations in family functioning more than clinical indicators, including neurological severity or child behavioral impairments (Bemister et al., 2015). Additional studies further support the profound impact of pediatric stroke on parenting roles and family cohesion. Labrell et al. (2018) described high parental stress, particularly in emotional and caregiving domains, with key stressors including uncertainty about the child's future, feelings of helplessness, and role strain. Lastly, Leal Martins et al. highlighted that parents of neonates with AIS experienced both acute and long-term emotional stress, compounded by perceived delays in diagnosis (Leal Martins et al., 2021). While child health outcomes were generally favorable, diagnostic uncertainty and the manner in which prognosis was communicated had a substantial effect on parental wellbeing, emphasizing the need for timely, clear, and empathetic communication in clinical care.

### 3.7 Psychological interventions and emerging therapies

#### 3.7.1 Evidence-based approaches

Addressing emotional and social functioning is essential in pediatric stroke, alongside motor and cognitive rehabilitation. Evidence for specific behavioral interventions in pediatric stroke is limited, but studies from adult stroke and broader pediatric acquired brain injury populations support integrated cognitive-behavioral and family-centered approaches (Wade et al., 2011, 2014; Lamond et al., 2022; Mrakotsky et al., 2022; Deepradit et al., 2023).

#### 3.7.2 Family involvement and telepsychology

Caregiver involvement is consistently identified as a key factor in improving both child outcomes and family dynamics. A systematic review on neonatal brain injury, including stroke, found positive effects in nearly 70% of studies evaluating early psychosocial interventions, such as parenting support, emotion regulation, and psychotherapy (Mrakotsky et al., 2022), highlighting the value of early, family-inclusive approaches. Two randomized controlled trials by Wade and colleagues further support the potential of online psychotherapeutic interventions (Wade et al., 2011, 2014). The first study evaluated Counselor-Assisted Problem Solving (CAPS), a CBT-based, therapist-guided program aimed at improving communication, emotional regulation, and problem-solving in adolescents with traumatic brain injury (TBI) and their families. CAPS participants showed significantly better functional outcomes, particularly in academic and work-related areas, compared to a control group using general online resources (Wade et al., 2011). Notably, benefits were greater among families with lower maternal education, emphasizing the need for accessible, guided interventions. In a follow-up study, Wade compared two versions of Teen Online Problem Solving (TOPS): one involving parents (TOPS-Family) and one for teens only (TOPS-TO; Wade et al., 2014). The family-inclusive model led to better improvements in executive functioning and externalizing behaviors, especially in low-stress environments, while the teen-only version was more effective under high-stress conditions. These findings align with recent studies on a telepsychological parenting skills program (I-InTERACT-North), involving 22 families of children aged 3–8 years with histories of neonatal stroke, Hypoxic-Ischemic Encephalopathy, and other neonatal conditions. The program included 7 online psychoeducational modules and 7 videoconference sessions with live therapist coaching, aimed at improving parenting skills and child behavior (Burek et al., 2021).

Despite the promising results of these studies, the limited data specifically focused on pediatric stroke highlights the need for further research to better understand the effectiveness of interventions in this population.

## 4 Discussion

While cognitive and motor outcomes following stroke in youth have been extensively studied, considerably less attention has been directed toward its psychological and, in particular, familial impact (Bemister et al., 2014, 2015; Yvon et al., 2018; Lehman et al., 2020; Li et al., 2021; Svensson et al., 2022; Janas et al., 2023; Rivella et al., 2023a). Pediatric stroke occurs during critical developmental stages when cognitive, emotional, and social functions are still undergoing maturation. The impact of age at stroke onset on long-term outcomes is complex and domain-specific. Children who experience stroke during the perinatal or neonatal period tend to exhibit lower functional and overall quality of life (Ghotra et al., 2015, 2018; Greenham et al., 2017a,b; Max et al., 2002). In contrast, strokes occurring at older ages are associated with slower social recovery and more pronounced emotional and behavioral difficulties, including symptoms of anxiety and depression (Greenham et al., 2017a,b; Max et al., 2002). These findings suggest that timing of stroke onset may interact with lesion severity and individual vulnerabilities to shape long-term psychological and social outcomes. Although typically focal in nature, brain injury can lead to widespread dysfunction, affecting not only motor systems but also emotional regulation, psychosocial adjustment, and overall QoL (O'Keeffe et al., 2012; Elbers et al., 2014; Greenham et al., 2017a,b, 2018; Lehman et al., 2020; Rohner et al., 2020). Internalizing symptoms, such as anxiety, depression, and post-traumatic stress, have increasingly been recognized as common and clinically significant consequences in pediatric stroke (Greenham et al., 2017a,b, 2018; Badar et al., 2020; Lehman et al., 2020). These emotional symptoms frequently co-occur with difficulties in peer relationships and social integration, reflecting the complex and multifaceted impact of the condition (Friefeld et al., 2011; O'Keeffe et al., 2012: Greenham et al., 2015; Smith et al., 2015; Camilleri et al., 2025). Psychological and social difficulties may persist even when motor and cognitive recovery appears favorable, suggesting a dissociation between physical outcomes and emotional wellbeing (Greenham et al., 2017a; Ledochowski et al., 2020). This divergence becomes particularly evident during the subacute and chronic phases post-stroke and may extend into adolescence or adulthood, often resulting in poorer functional outcomes, lower economic independence, lower life satisfaction and mental health burden (Hurvitz et al., 2004; Szabados et al., 2023). The emotional and social consequences of pediatric stroke align with a broader pattern seen in other neurological disorders, though each condition presents distinct features that shape the psychosocial profile of affected children. Children with brain tumors often experience heightened levels of depression and anxiety, linked both to the tumor itself and the neurocognitive effects of treatment (Zeltzer et al., 2009; Brown et al., 2023). In pediatric epilepsy, unpredictable seizures contribute to anxiety, social withdrawal, and reduced self-esteem, while survivors of traumatic brain injury frequently show long-term emotional and behavioral difficulties, including social impairments (Li and Liu, 2013; Kwong et al., 2016; Jones et al., 2021).

The persistence of emotional and social difficulties in pediatric stroke may be explained by both neuroanatomical and psychological factors. Severity of neurological impairment at stroke onset is a strong predictor of both emotional and social outcomes: children with more extensive deficits or involvement of multiple vascular territories exhibit higher internalizing symptoms and lower social functioning (Anderson et al., 2014; Bulder et al., 2011; Ryan et al., 2021). Early stroke and larger lesion size are associated with more pronounced deficits in executive function, emotional regulation, and peer interactions (Greenham et al., 2017a,b; Ghotra et al., 2015; Anderson et al., 2014; Lo et al., 2020). Lesions may involve regions critical for emotion processing and social cognition, such as the basal ganglia, thalamus, orbitofrontal and prefrontal cortices, temporal pole, and amygdala (Beauchamp and Anderson, 2010; Rivella et al., 2023a). Injury to these cortico-striatal-limbic circuits and components of the social brain network can impair executive function, emotional regulation, and interpersonal behavior (Blanken et al., 2017; Patwardhan et al., 2021). The impact of such disruptions may not be immediately evident. The “growing into deficit” hypothesis suggests that the effects of early brain injury become more pronounced over time, particularly during adolescence, when the developmental emergence of higher-order cognitive and socio-emotional functions places increased demands on neural systems previously compromised (Barker et al., 2010). These neurodevelopmental vulnerabilities are further exacerbated by factors such as prolonged hospitalization, interruption of age-appropriate experiences, disease-related symptoms and loss of previously acquired skills, which contribute to early emotional distress and long-term psychosocial risk (Pinquart, 2014; Berkelbach van der Sprenkel et al., 2022; Fardell et al., 2023; Wallace et al., 2025). Environmental and social contexts also play a crucial role in shaping psychological outcomes. Experiences of stigma, reduced opportunities for participation, and limited peer interaction, often associated with ongoing physical or cognitive disabilities, can restrict social development and reinforce feelings of isolation (Blom et al., 2003; Martinez et al., 2011; Anderson et al., 2014; O'Keeffe et al., 2017; Berkelbach van der Sprenkel et al., 2022). Such barriers limit engagement in typical activities, increase the risk of internalizing disorders, and contribute to diminished self-esteem, further compounding the psychosocial burden in pediatric stroke survivors (Spilt et al., 2014; Hosokawa and Katsura, 2017; Camilleri et al., 2025). The effects of pediatric stroke extend beyond the affected child, substantially impacting parents and the broader family system. When a child is suddenly diagnosed with a serious medical condition, parents are often catapulted into intensive caregiving roles with little warning or preparation (Soufi et al., 2017). They must quickly comprehend complex medical information, manage multidisciplinary care, and advocate for their child's needs within healthcare and educational systems (Raina et al., 2004; Smith et al., 2022). These demands are often heightened in pediatric stroke, where urgent medical decisions must be made in the context of diagnostic ambiguity and rapidly evolving clinical scenarios (Soufi et al., 2017; Khan et al., 2022). The sudden and unpredictable onset of stroke commonly triggers acute emotional responses in caregivers, including shock, fear, and uncertainty (Leal Martins et al., 2021; Smith et al., 2022). Caregivers are frequently faced with distressing images of neurological deterioration and uncertain prognoses, which may generate intense psychological strain (Khan et al., 2022). This emotional burden is compounded by the need to manage long-term rehabilitation and adapt to potentially life-altering functional outcomes (Soufi et al., 2017). Communication at the time of diagnosis plays a central role in shaping caregiver responses. Studies from general pediatric care settings have shown that clear, empathetic, and timely communication is associated with lower levels of caregiver distress and increased trust in medical teams (Solan et al., 2018; Lee et al., 2020; Ciurria et al., 2025). In contrast, experiences marked by delayed, vague, or insensitive communication can undermine parental confidence and increase psychological vulnerability (Leal Martins et al., 2021; Peterson et al., 2021; Khan et al., 2022). Caregiver distress has significant implications for child outcomes. Parental psychological functioning, and in particular maternal mental health, has consistently been identified as a robust predictor of children's emotional and behavioral adjustment following stroke (Greenham et al., 2015; Peterson et al., 2021; Rivella et al., 2023a). When caregivers experience high levels of stress, anxiety, or depressive symptoms, parental behaviors may be disrupted, emotional availability may be reduced, and family functioning can become strained (Ribas et al., 2024). These dynamics contribute to increased emotional and behavioral problems in children and may interfere with recovery trajectories. Despite the significant impact of emotional wellbeing on recovery and overall quality of life following pediatric stroke, psychosocial interventions remain limited. Given the close connection between caregiver and child adjustment, integrating psychosocial support into pediatric stroke care is crucial. Multidisciplinary, family-centered care that addresses both neurological recovery and emotional dynamics is critical to optimizing long-term outcomes (Mrakotsky et al., 2022). Routine psychosocial monitoring, including caregiver wellbeing, could improve clinical outcomes and strengthen family resilience. While research specific to pediatric stroke is limited, interventions for pediatric acquired brain injury, such as family-centered cognitive-behavioral therapies, have proved promising in enhancing emotional regulation and family functioning (Wade et al., 2011, 2014, 2018; Burek et al., 2021; Mrakotsky et al., 2022).

This review critically examines pediatric stroke outcomes, with particular emphasis on the psychological sequelae in children and their impact on parents, an area that remains under-investigated (Kolk et al., 2011; Malone et al., 2022; Baak et al., 2023). One notable strength of this review is its comprehensive synthesis across emotional, behavioral, and social domains, allowing for a more integrated understanding of recovery than previous studies. By addressing emotional, behavioral, and social dimensions, it offers a refined understanding of recovery, shaped not only by neurological factors, developmental stage, and environmental context, but also by the timing and severity of the initial injury, which may predict psychosocial and family outcomes (Anderson et al., 2014; Ghotra et al., 2015, 2018; Greenham et al., 2017a,b; Max et al., 2002; Ryan et al., 2021). The family environment emerges as a pivotal determinant of post-stroke adjustment. The inclusion of parental psychological outcomes as a focus further enhances the review's contribution, highlighting how anxiety, depression, or feelings of guilt in parents consistently predict children's emotional and behavioral outcomes. This underscores the imperative to incorporate parental mental health within pediatric stroke care frameworks. From a clinical perspective, the early identification of psychological symptoms in both children and caregivers is essential to facilitate timely intervention. The review effectively translates evidence into actionable recommendations, emphasizing family-centered, multidisciplinary approaches that integrate psychological support, neurorehabilitation, and educational planning to promote emotional resilience and social reintegration (Wade et al., 2011, 2014, 2018; Burek et al., 2021; Mrakotsky et al., 2022). Additionally, schools play a vital role by implementing inclusive practices, offering emotional support, and providing teacher training to enhance understanding and facilitate participation (Proios et al., 2022). By combining a broad literature synthesis with practical clinical and educational insights, this review provides a robust foundation for improving pediatric stroke care.

## 5 Limitations

Despite these strengths, several methodological limitations should be considered. Studies vary widely in stroke subtype, lesion characteristics, age at onset, assessment tools, and timing of evaluations, which limits comparability and generalizability (Table 1). Inconsistencies in study design and outcome measures add further complexity. Differences between parent-, teacher-, and self-reports also introduce potential bias, as emotional and behavioral problems may be perceived differently across informants. Moreover, child self-reports can be constrained by developmental limitations, especially in younger children, reducing the reliability of psychosocial assessments. These issues highlight the need for multi-informant approaches to capture a more accurate and comprehensive understanding of psychological functioning. Small sample sizes, cross-sectional designs, and the frequent absence of appropriate control groups further reduce the reliability and robustness of findings. Most studies are also based in Western, high-income countries, with limited consideration of sociocultural and economic contexts. Yet, extensive evidence shows that socioeconomic status (SES) significantly influences child health and development and access to rehabilitation and educational services (Poulain et al., 2020; Bartha-Doering et al., 2021; Sun et al., 2023; Rony et al., 2024). Lower SES is also associated with reduced access to specialized care, delays in diagnosis, and fragmented neuropsychological follow-up, all of which may compound post-stroke difficulties (Sun et al., 2023; Rony et al., 2024). Beyond SES, family dynamics and cultural factors, such as parenting practices, beliefs about disability, and stigma, play a critical role in shaping access to care, treatment adherence, and social participation. However, such variables are rarely incorporated into pediatric stroke studies, limiting our understanding of how environmental and familial contexts interact with neurological injury. Their systematic inclusion is essential to ensure equitable, personalized models of care and to guide educational and rehabilitative planning that reflects the diverse realities of affected families. Finally, the integration of neurobiological, psychological, and social perspectives remains limited, hindering a comprehensive understanding of the complex mechanisms underlying post-stroke adjustment. Future research should adopt longitudinal, multidisciplinary, and culturally sensitive designs, expand beyond Western contexts, and include family- and context-specific variables to support the development of ecologically valid, family-centered interventions for children and their caregivers.

## 6 Conclusion

Pediatric stroke leads to profound and long-lasting consequences that go far beyond motor and cognitive deficits. Emotional wellbeing, social development, and family dynamics are often significantly disrupted. The long-term outcomes are shaped not only by the neurological severity of the stroke itself, but also by the psychological health of caregivers and the overall functioning of the family unit. Despite increasing recognition of these challenges, psychosocial needs are often insufficiently addressed in standard care. A multidisciplinary, family-centered approach that integrates psychological support is essential to comprehensive recovery. Future research and clinical practice should consider the development and implementation of accessible, evidence-based interventions aimed at promoting resilience and enhancing long-term QoL for both patients and their families.
